# Child Birth Practices and Utilization of Antenatal Care (ANC) Services Among Migrant Tribal Women in Urban Areas of Gujarat

**DOI:** 10.7759/cureus.39363

**Published:** 2023-05-22

**Authors:** Niraj Pandit, Vruddhi Patel

**Affiliations:** 1 Community Medicine, Sumandeep Vidyapeeth Deemed to be University (SVDU), Vadodara, IND

**Keywords:** vulnerable population, accredited social health activist (asha), tribal women, home birth, qualitative study

## Abstract

Introduction: Tribal women constitute a vulnerable population and migratory tribal women living in urban areas are among the most vulnerable and neglected sections. The current study was conducted among migratory tribal women living in the urban areas of Gujarat to understand their antenatal care (ANC) and child birth practices.

Methodology: This was a community-based mixed methods study, conducted during 2022, in four major cities of Gujarat. The sample size for the quantitative study consisted of 592 participants. Inclusion criteria for participants were tribal women migrants to urban areas; migration for employment; less than a year of residence in the urban area; married women; and working on construction-sites. The qualitative study included 20 tribal women selected from cities and a total of 24 grassroots workers and in-depth interviews were conducted.

Results: The participating women were in the age group of 16-43 years, with mean age being 26 years. Almost 67 (11%) women were pregnant at the time of the study. Around 51% of the women had FOUR antenatal care (ANC) visits during their previous pregnancy. Around 63 (18%) women had home births. Qualitative data revealed that their deep-rooted cultural practices and beliefs influenced their ANC patterns, child birth practices, and utilization of hospital services.

Conclusion: Migrant tribal women are considered a vulnerable population in urban areas, as they do not have local documents. Further, they are bound by deep-rooted cultural beliefs. There is a need to use technology for developing tracking systems, in order to provide better maternity care to these women.

## Introduction

Tribal women in India constitute a significant portion of the country’s population and are an integral part of the country’s diverse culture heritage. They play a vital role in the preservation and continuation of their traditional customs and practices, which are deeply rooted in their communities. According to the 2011 census, tribal women constitute 8.6% of India’s total and 7.2% of Gujarat's total female population [1]. They are primarily concentrated in the country’s northeast, central, and southern regions, with the highest concentrations found in the states of Chhattisgarh, Jharkhand, Odisha, and Madhya Pradesh [2-3].

Migrant workers are predominantly employed in the construction sector. They usually belong to the states of West Bengal, Bihar, Chhattisgarh, Madhya Pradesh, and Rajasthan [4]. Rates of participation in manual labor were found to be highest among the scheduled tribes (ST) population; they stood at 49.1%, compared to 30.3% for the general population. Tribals in India face insurmountable challenges due to their poor socio-economic conditions, poverty, unemployment, displacement, lack of opportunities, low accessibility, and poor awareness of government programs. This results in large-scale migration of rural tribal populations to urban areas in search of livelihoods, either temporarily or on a permanent basis [5].

With rapid urban growth over the past two decades, the construction sector has emerged as one of the largest employers of labor in the country, employing close to 50 million people. Approximately 30% of this labor force consists of female workers, most of whom engage in informal work. The construction sector relies heavily on short-term migrant workers, offering dual work opportunities to both female and male workers. Female workers are often considered to be ‘helpers’ or ‘less skilled’ due to which they receive less than minimum wages, despite engaging of long hours of laborious and hazardous work [6].

Tribal women and children also are associate migrants with their male partners or parents. Their health, particularly during pregnancy and child birth, poses a major challenge during migration to a new city or town.

The national health indicators are gradually improving for maternal and child health. As per National Family Health Survey-5 (NFHS-5), in the state of Gujarat, almost 76% of pregnant women had four antenatal care (ANC) visits and 92% of deliveries were institutional. Out of the total number of institutional deliveries, 64.3% were conducted at private hospitals, whereas 43.3% deliveries were conducted at public health institutions. It was also reported that 60% of ANC mothers had consumed iron and folic acid (IFA) tablets [7].

However, migrant tribal women still face major ANC and child birth challenges, particularly while living in urban areas. There is limited information available on this. The current study aimed to understanding the status of ANC patterns and child birth practices among migrant tribal women residing in urban areas.

## Materials and methods

This was a community-based mixed methods study. It is part of larger study on reproductive and child health issues among migrant tribal women living in the urban areas of Gujarat, India. The present study was conducted in Surat, Rajkot, Ahmedabad, and Vadodara, the four most populous municipal corporation areas in the state of Gujarat.

The sample size for this study was calculated by considering the utilization of birth control measures by migrant tribal females, a principal outcome variable of our previous study [8]. The calculation was as follows: 27% birth control utilization rate, at 95% confidence interval, 80% power, 20% permissible error, and 2% design effect.

The final sample size consisted of 580 participants; this number was divided among four cities; hence, the sample size from each city came to around 148. Based on the feasibility issue the 592 participants enrolled for final data collection. Each of the cities were divided arbitrarily in to four equal quadrants on the map. In each quadrant, the study team identified as per the inclusion and exclusion criteria women living with their families. With simple random sampling method, the desired sample size was achieved. 

Inclusion criteria for participants were as follows: tribal women migrants to urban areas; migration for employment purposes; less than a year of residence in the urban area; married women in the reproductive age group; and working on construction sites. Exclusion criteria were as follows: unmarried tribal women, as well as those who had been residing for more than a year in the urban area and had permanent local addresses.

The qualitative part of the study included 20 tribal women (excluded from the quantitative study) selected from the four cities and a total of 24 key informants or grassroots workers [eight Auxiliary Nurse and Midwife (ANMs) and 16 Accredited Social Health Activist (ASHA) workers]. In-depth semi-structured interviews were conducted with these women and with the grassroots workers, in order to understand the service gaps related to the study population.

The study was started after obtaining permission from the institutional ethics committee. The Sumandeep Vidyapeeth Institutional Ethics Committee has approved the study with reference number SVIEC/On/Medi/RP/20027. For data collection, each city was divided into four equal parts using the local and google layered map. Each quadrant was mapped to identify the construction sites. They trained the data collection team which was consisted of three and two were females. The team visited each city. They demarcated four quadrants on ground in each city. Next, the team has marked the participants in each quadrant to recruit the participants for the study. Women living in the selected areas, meeting the inclusion criteria were recruited for the study. The quantitative component was conducted using a pilot pre-tested questionnaire. The survey questionnaire included details about socio-demographic characteristics, pregnancy history, utilization of health services, as well as participants’ history of child birth and ANC. The trained project staff visited four cities and collected data at the place of work of participants. On-site permission was taken from the construction contractor. Data collection was carried out, after obtaining informed consent from the participants.

The qualitative component of the study involved in-depth interviews of migrant tribal women excluded from the quantitative survey. These interviews were conducted using semi-structured pilot pre-tested questionnaires along with a pre-tested guide. The in-depth interviews were audio recorded with the prior consent of the participants, in order to gather detailed information. The open-ended questions dealt with their current status of child birth, to gain an understanding of the cultural and social factors influencing child birth practices and health-seeking behavior among tribal women. Data were collected throughout the period from April 2022 to December 2022.

The quantitative analysis was carried out using Microsoft Excel. For the qualitative part, interviews were recorded and transcribed in the local language, and the transcripts were analyzed thematically and presented in a comprehensive manner. The results of the study were presented using thematic descriptive analysis, wherein key issues relating to ANC services and child birth practices were highlighted.

## Results

The current study recruited women 592 for the quantitative study. Table 1 summarizes the socio-demographic characteristics of the participants. Participating women were aged between 16 and 43 with the majority being between the ages of 21 and 25 (42%) and the mean age was 26 years. Approximately 60% of the total participants were illiterate. Further, 88% participants belonged to nuclear families, whereas 51% had upper middle class economic status.

**Table 1:** Socio-demographic details of participants.

Age of participants	N	Percentage (%)
16-20	127	21.5
21-25	246	41.6
26-30	101	17.0
>31	118	19.9
Education		(%)
Illiterate	354	59.8
Primary	136	22.9
Secondary	75	12.7
Higher secondary and above	27	4.6
Family Type		(%)
Nuclear	521	88
Joint	71	12
Socio-economic status (modified Prasad Classification)		(%)
Upper class	212	35.8
Upper middle class	305	51.6
Middle class	70	11.8
Lower middle class	5	0.8
Lower class	0	0
Total	592	100

Table 2 depicts information about the participants’ pregnancy status. All the participants were categorized based on their pregnancy status. A total of 67 women (11.3%) were pregnant at the time of the study; among these, 21 (31%) had conceived for the first time, whereas 46 (69%) were cases of multigravida pregnancy. Non-pregnant women were classified into three categories: women with one or more children (441; 74.5%); women who had conceived once but had no living child (8; 1.4%); and women who had not yet conceived (76; 12.8%).

**Table 2:** Current pregnancy and child status of tribal women under study (N=592).

Currently pregnant (N=67)	N	(%) of 592
Primigravida	21	3.5
Multigravida	46	7.8
Currently not pregnant (N=525)	N	
No conception till time	76	12.8
Women who conceived once but not having any child (abortions, miscarriage, or child death)	8	1.4
Women with one child or more women with child between 0 and 5 years were 355	441	74.5
Women with child present between 0 and 5 years	(N:355)	% of 355
Women with child between 1 and 5 years	281	79.2
Women with child present 1 year or less than 1 year	66	18.6
Women with child death after delivery	8	2.2

Table 2 also gives information about women having children in the age group of 0-5 years. A total of 355 women had at least one child in the age group of 0-5 years.

Table 3 depicts the last delivery status. A total of 355 (60%) women had at least one child aged between 0 and 5 years. About 18% of deliveries had occurred at home. It was found that majority of the deliveries or home births (56; 16%) were conducted by the traditional Dai, whereas two home births (0.5%) were carried out by the mothers themselves without any help. Ninety-six per cent of the total number of deliveries were vaginal deliveries.

**Table 3:** Last delivery place and related factors distribution.

Place of delivery	n (%)
Hospital	292 (82%)
Home	63(18%)
Total	355
Details of last child delivery	n (%)
Hospital delivery	292 (82%)
Delivery by doctors	89 (25%)
Delivery by nurse	202 (57%)
Home delivery	63 (18%)
Delivery by relative	6 (1.5%)
Self delivery	2 (0.5%)
Delivery by traditional Dai	56 (16%)
Delivery Type	n (%)
Vaginal delivery	341 (96%)
C-section	14 (4%)
Total	355

Table 4 explains ANC service utilization among the migrant women. The findings suggest that a good number of women (177; 50%) had four or more than four antenatal visits, whereas 2.8% women did not have any antenatal check-ups during their pregnancies. The reasons for not taking antenatal check-ups were lack of accessibility to and awareness about healthcare services. Almost 80% of these migrant women opted for government health facilities for antenatal check-ups. It was found that 63% women had consumed one IFA tablet per day during the last 6 months of their pregnancies; however, 36.6% had not consumed any IFA tablets. Further, 99% of these women had received tetanus toxoid (TT) vaccination during their pregnancies.

**Table 4:** Last delivery ANC practices among tribal women who has child 0-5 years. ANC, antenatal care; IFA, iron and folic acid; TT, tetanus toxoid

Key ANC events		N:355
Antenatal check-up during pregnancy period	0 visit	10 (2.8%)
	1-3 visit	168 (47.3%)
	4 visits	36 (10.2%)
	5-8 visits	67(18.8%)
	9 visits	74(20.9%)
From where ANC taken	Government hospitals	285 (80.2%)
	Private hospital	60 (17%)
	Not taken	10 (2.8%)
IFA tablets consumptions	Consumed 1 IFA per day for last 6 months of pregnancy	225 (63.4%)
	Not consumed	130 (36.6%)
Mother’s vaccination (TT)	Vaccinated	352 (99.2%)
	Not vaccinated	3 (0.8%)
Reason for not taking ANC	Lack of accessibility	5 (50%)
	Lack of awareness	5 (50%)

The current study found that most of the women returned to their hometowns for their deliveries. Here, they had followed a common practice in Hindu tribal culture.

Qualitative analysis based on themes

Antenatal Care: Delay in the Early Registration of Pregnancy

In the current study, all participants were Hindu tribal women. According to them, as part of tribal cultural beliefs, women could not reveal pregnancy during the early weeks. They believed that doing so would lead to unfavorable outcomes. This cultural belief posed a major hurdle in the early registration of pregnancy.

Response 1: “My mother in-law told me not to reveal the pregnancy during the first few weeks; it is considered unlucky in our culture.”

Response 2: “We are not allowed to disclose the pregnancy yet; so I will not venture outside for the registration.”

Similar responses were received from a majority of the tribal women. Even ASHA workers reported the same in their interviews.

ASHA Response 1: “The participant does not come outside even to talk; only the family members respond on behalf of the pregnant lady. So we are unable to get an early registration; even if we know that the woman is pregnant, we are unable to talk to her.”

Response 2: “If we ask them about their last date of menstruation, women lie to us, because they do not want to reveal the pregnancy during the first few months. Generally, they reveal their pregnancy from the fourth month onwards; due to this, we are unable to make an early registration of pregnancy.”

Frequent migration also poses a key challenge to ANC. Despite frequent migration, the current study reveals that almost 50% of the women made four or more ANC visits at health facilities, and only 10 women (2.1%) did not avail any ANC visits. This is a quite satisfactory number for migrant workers. Though these women do not have permanent local addresses, they have a good rapport with the urban health centre and ASHA workers.

Responses from ASHAs, as providers of ANC services, revealed strong positivity among young women regarding the health of mother and child.

Response 1: “These women are uneducated yet sensible enough to understand the benefits of ANC; if we explain things in detail, they trust us. We are in constant contact with them.”

Response 2: “In some tribal groups, their families are very rigid; but the newlywed couple or young mothers are more practical now-a-days. Sometimes, these mothers even lie to their in-laws if they are more rigid and do not allow them to go for regular check-ups. All newlywed couples are more understanding now-a-days; if something is good for both mother and child, they are ready to avail the service. Young husbands are more supportive and concerned; they come to the urban health centre and follow the doctor’s instructions.”

Delivery Practices: Home Birth Rates (18%) Higher Than National Average

This study found unspoken emotions and comforting behavior among tribal women about childbirth at different places. For instance, giving birth at home in the presence of a known person or relative, rather than in a hospital in an unknown hand was felt to be more comfortable. They also felt that traditional birthing practices were more effective in reducing pain and promoting a smooth delivery. Majority of the women reported discomfort in a hospital environment. Their statements are recorded below.

Response 1: “I have two kids; both the deliveries happened at home, because we are afraid to go to doctor. I have heard they do not let any family member enter the labour room; so, what if they do an operation instead of normal delivery? I was scared; so, I did not go to the hospital for delivery.”

Response 2: “The delivery of my first son was carried out at home; there is a lady, she performed my delivery. Doctors had told me that my blood cell count was very low, but that lady performed my delivery normally and without any extra pain. And I went to the doctor for the delivery of my second child; the doctor checked me once, and thought, ‘Let me cut this lady up.’ So, he operated on me and that was painful; so, I feel home delivery is good, because doctors do operations for money.”

A few women reported self-delivery. They opined that the reason may be their intense daily physical labor. Their bodies were more flexible and stronger, which led to a normal, vaginal, pain-free delivery. A few women reported that they did not feel labor pains before the birth occurred; thus, sudden childbirth occurred while they did construction work or regular household chores and no one was present around.

Response 1: “My delivery took place when I was at my village; there is a Daima in the village. But on that day, I was alone at home, no one was at home to call the Daima. I was doing regular chores at home, felt a little pain, and suddenly the baby was born. Then, my husband came home; he cut the cord; and then we both went to the PHC for registration.”

## Discussion

The ANC service is the key service indicator for maternal and child health. Good maternal ANC service converts to institutional delivery in majority of the cases. The same conclusion was reached by an Ethiopian systematic review meta-analysis published in 2018 [9]. Thus, ANC care is crucial for maternal health as well as childbirth practices and child health. Tribal migrant women constitute the most vulnerable group in the population. They need special care. The current study reveals that almost 70% of migrant tribal women were young, aged less than 30 years, and around 60% were illiterate. However, their socio-economic conditions were quite good, with less than 1% belonging to the lower middle class and lower class. Similar observations were reported by a study among the Kashmiri tribal population [10] and by a study on the Andhra Pradesh tribal population [11]. It was observed that 67 (11%) women were pregnant at the time of interview, and nearly 60% of women had children in the age group of 0-5 years. This study evaluated the ANC and childbirth practices among these women.

A minimum of four ANC visits is the quality indicator for the maternal health program [12]. This study observed that almost half of the women had made four ANC visits to the hospital or health center. This percentage is lower than the national average of 58% [13] as per NFHS-5 survey and lower than the Gujarat state average of almost 77% [6]. Thus, the ANC coverage was low, but it was still higher than that of the DLHS-3 survey of 2007-08, wherein ANC coverage was just 4%-14% among tribal women in Madhya Pradesh, Rajasthan, Odisha, and Chhattisgarh [14]. Though the quality of services has improved over time, gaps are still observed. The current study reveals various gaps in services for the tribal migrant population living in urban areas, through qualitative in-depth interviews.

The cultural practices of the tribal groups posed a major obstacle in ANC. Early registration of pregnancy was not accepted by this community. They believed that early disclosure of pregnancy would harm the unborn child. They still believed in traditional healers and the local Dai. C. J. Sonowal has also reported such deep-rooted beliefs among pregnant tribal women, in his study [15]. Such customs need to be rectified with scientific facts. Also, the new generation living in urban areas can change such practices with the help of frontline workers, such as ASHAs or ANMs. A study from Kerala also reported that early registration is the key to receive full ANC and effectively utilize maternal and childbirth services [16].

Coverage by other ANC services, such as two doses of the TT vaccine was observed at almost 99%, which is higher than the national average (92%) and state average (89%). Similarly, IFA tablet consumption rates stood at 63% among participants; this is higher than the national average (26%) and the Gujarat state average (43%) [7,13]. These indicators reflect the role of local healthcare worker in these cities. They have been working proactively for such vulnerable groups of women. If the government works likewise, ANC coverage shall definitely improve greatly.

The lack of local documentation also poses a major challenge for these women. Similar facts were revealed in a study conducted in Odisha by Contractor et al. [17]. These women do not possess local documentation such as Aadhar cards or proofs of address. This becomes a major limitation for the government health system to incorporate them in its services. In Gujarat, the government health system has tried to incorporate this population; however, the mechanism for transferring-out to health systems of other states is not well established. This constitutes a major hurdle in the tracking of ANC as well as health of mother and child.

The current study found that home birth rates are still high, up to 18%, in this group. This is quite high as compared to the national average of 3.2% home births by skilled health personnel and the Gujarat state average of 1.6% home births [7,13]. The qualitative analysis has depicted that tribal women have no confidence in the healthcare system. They were scared of hospitals and doctors. Similar findings were observed by Contractor et al.’s study [17]. The reasons included low trust in hospital services, poor accessibility to hospitals, fear of surgery, difficulties in accessing benefits of government schemes, and so on. Therefore, the system needs to think in a simple yet innovative manner to reach this unique group.

The most striking finding of this study is the mismatch between the utilization of grassroots worker services and hospital services. This vulnerable population has confidence in ASHAs and ANMs who are close to them, but not in the hospital services. Policymakers need to view this issue from the patient’s perspective. The healthcare system can use technology to improve the confidence of the client in hospital services.

## Conclusions

First, this study revealed that migrant tribal women constitute a unique and vulnerable population. They do not have government records at the local city level where they work. Second, they reside in the city for a short duration. Third, they move back to their hometowns for delivery and child care. They have confidence in the services of grassroots-level workers, such as ASHAs, but not in hospital services. Community to hospital services gap was found and this is required to bridge. In addition, there is a need to develop technology-based ANC and child care systems linked with Aadhar numbers. ASHAs, in collaboration with the local municipal corporation health system and construction-site employers, should develop a system of recording the health status of all migrant workers, especially the maternal health of migrant women workers. Thus, there is a need to develop triangulation of information-sharing system among the local health service, the state-of-origin health service, and employers which will help effective provision of essential ANC and childbirth services.
